# Reply to “Comment on ‘Intrinsic Formamidinium Tin Iodide Nanocrystals by Suppressing the Sn(IV) Impurities’”

**DOI:** 10.1021/acs.nanolett.5c03114

**Published:** 2025-07-11

**Authors:** Dmitry N. Dirin, Alexander Wieczorek, Sebastian Siol, Maksym V. Kovalenko, Maryna I. Bodnarchuk

**Affiliations:** † Institute of Inorganic Chemistry, Department of Chemistry and Applied Biosciences, 31064 ETH Zürich, CH-8093 Zürich, Switzerland; ‡ 28501 Empa−Swiss Federal Laboratories for Materials Science and Technology, CH-8600 Dübendorf, Switzerland

## Abstract

In this reply, we address the comments of A. Dhingra on our prior work titled “Intrinsic Formamidinium Tin Iodide Nanocrystals by Suppressing the Sn­(IV) Impurities”.

We appreciate the interest shown by A. Dhingra (10.1021/acs.nanolett.5c02296) in our work titled “Intrinsic Formamidinium Tin Iodide Nanocrystals by Suppressing the Sn­(IV) Impurities”.^1^ Constructive dialogue is essential to scientific progress. However, several claims raised in their comment are, in our opinion, factually inaccurate or based on misinterpretations of our original paper. We are glad to provide a point-by-point response to their comment and explain how the XPS analysis in our original article not only adheres to established best practices in the field but also offers a highly robust investigation of the Sn chemical state via Auger parameter analysis. This workflow is highly suited for semiconducting and insulating materials and circumvents typical pitfalls in the analysis of the Sn chemical state.

The points raised by A. Dhingra are summarized below, together with our respective responses:

1. In the abstract, A. Dhingra suggests “to rectify the fits to the high-resolution X-ray photoelectron spectroscopy (XPS) raw data for the S 2p core level”.
**Response:** We cannot comment on the claim that our S 2p core level fits need to be rectified, as we did not even report such fits. We would like to point out that our samples do not contain any sulfur. This can be observed in our reported survey spectra shown in Figure XPS1 in the Supporting Information of our original paper. We note that the abstract by A. Dhingra appears to be identical to a previous comment of theirs, which was provided to an entirely different paper, see ref 2.
2. A. Dhingra further notes that the separation of the Sn^2+^ and Sn^4+^ peaks is reported to be more than 0.5 eV. Based on this, they suggest fitting the Sn 3d core level for chemical state analysis.
**Response:** A. Dhingra suggests fitting the Sn 3d core-level spectra for chemical state analysis. Most importantly, the Sn 3d region shown in Figure 1d of our original paper exhibits perfectly symmetrical peaks, which indicates that the material predominantly exhibits a singular oxidation state. This necessitates the assignment of the chemical state based on absolute binding energies. While there are indeed selected studies suggesting a separation of more than 0.5 eV between Sn^2+^ and Sn^4+^ core-level peaks, meta-analysis of the NIST database reveals that there is a rather large spread in the reported binding energy values for the Sn 3d_5/2_ core levels of both SnO (486.5 ± 0.5) eV and SnO_2_ (486.8 ± 0.4) eV.^3^ These margins of error, calculated from the respective standard deviations, are beyond the expected binding energy difference between SnO and SnO_2_. This highlights the ambiguity of using absolute Sn 3d core-level binding energies to determine the Sn chemical state. This is a well-known and well-reported problem in the community.
3. The presence of surface charging effects is questioned by A. Dhingra. Additionally, they suggest surface charging would amplify the peak separation between the Sn^2+^/Sn^4+^ species as the Sn^4+^ would be present on the sample surface.
**Response:** Surface charging is a known effect for semiconducting and insulating samples, as previously reported by Baer et al.^4^ In addition, surface photovoltages generated during photoelectron spectroscopy have been reported to induce additional nonchemical shifts of 0.7 eV for metal halide perovskites^5^ and other semiconductors.^6^ Additionally, one has to differentiate between differential and static charging.^7^ Only differential charging would change the observed difference between Sn^2+^/Sn^4+^ Sn 3d core-level binding energies and only if Sn^4+^ was present predominantly on the sample’s surface. In our original paper, the Sn^4+^ impurities stem from precursor impurities, and the samples were transferred under inert-gas conditions to the XPS device. As a result, the Sn^4+^ impurities could be distributed across the whole nanocrystal volume and not only on the nanocrystal surface. If Sn^4+^ was located mainly on the surface, differential charging, if present, would make relying on absolute binding energy separation even more problematic. For all the reasons given above, performing chemical state analysis based on the Sn 3d core level alone, especially on the basis of absolute binding energy values, is not feasible and can lead to erroneous and irreproducible results.
4. A. Dhingra claims it would be “evident that the 3d_5/2_ and the 3d_3/2_ XPS peaks of the Sn 3d core level were fitted separately” in our work.
**Response:** There is no indication in our reported Sn 3d core-level spectra to support A. Dhingra’s suggestion that the 3d_5/2_ and 3d_3/2_ peaks were fitted independently. Instead, as evident from Figure 1d of our original paper, we constrained the relative peak area to a 3:2 ratio as expected from spin–orbit splitting, maintained the same peak full-width-half-max (fwhm), and constrained the Sn 3d_5/2_ and Sn 3d_3/2_ peak distances to 8.41 eV based on literature reports.^8^
5. To “solidify the claimed surface chemistry”, A. Dhingra suggests presenting detailed Voigt fits to the raw core-level photoemission spectra of the involved atomic species.
**Response:** We decisively abstained from making claims about the exact surface stoichiometry of the samples due to the complex core–shell structure of the investigated nanocrystals. Instead, we only highlighted the increase of the formamidinium (FA^+^) relative to the oleylammonium (OAm^+^) features in the N 1s core-level region with increased probing depth in Figure 1f.
6. A. Dhingra refers to the works of Greczysnki et al. to highlight the invalidity of the C 1s charge referencing for chemical state analysis.
**Response:** We are well aware of the reports by Greczynski et al. criticizing the usage of C 1s referencing for chemical state analysis due to its dependency on the sample’s work function.^9^ Biesinger provides a less pessimistic view on the topic with typical inaccuracies resulting from C 1s referencing on the order of ± 0.25 eV.^10^ In any case, it is clear that the lack of a well-defined charge reference adds to the uncertainty in the reported absolute binding energies. We fully agree with this notion, which is exactly the reason why we do not interpret absolute binding energies in this paper, which we would have to do when relying on the Sn 3d feature alone.
  Instead, we make use of the Auger parameter (AP) to investigate the Sn chemical state. The AP is insensitive to surface charging as well as an erroneous referencing of the energy scale. In addition, the AP often exhibits much larger shifts than the corresponding core-level binding energies. AP analysis is well established for chemical state analysis of Sn-based compounds.^11–13^

  This also motivated earlier work by our coauthors Wieczorek and Siol on the robustness of the AP for analyzing Sn-based halide perovskites.^14^ In this study, the modified AP α′ of Sn is calculated according to
$$\alpha^{\prime }=E_{\mathrm{kin}}\left(\mathrm{Sn}M_{4}N_{4,5}N_{4,5}\right)+E_{b}\left(\mathrm{Sn}3d_{5/2}\right)$$ 1
  where *E*_kin_(Sn M_4_N_4,5_N_4,5_) denotes the kinetic energy of the Sn M_4_N_4,5_N_4,5_ Auger emission feature maximum, and *E*_b_(Sn 3d_5/2_) denotes the binding energy of the Sn 3d_5/2_ core-level maximum.
Figure 1A shows a Wagner plot for a large number of different Sn-based compounds. Despite the different chemistries, the observed range in *E*_b_(Sn 3d_5/2_) core-level binding energies is rather small (<1 eV). In contrast, the spread in the AP is significantly higher. In addition, we find an improved accuracy and precision of the AP for chemical state analysis. For instance, we find a range of 0.6 eV for *E*_b_(Sn 3d_5/2_) core-level values of ASnI_3_-type perovskites. For the resulting modified Auger parameter α′, this range was diminished to <0.1 eV. This agrees with the similarly expected nearest neighbor interactions of Sn in ASnI_3_-type perovskites, which dominate the Sn chemical state. We obtained similar results between ASnBr_3_-type perovskites, with a value of α′ distinct from ASnI_3_-type perovskites, which can be likewise explained by the nearest-neighbor interactions. Crucially, as depicted in Figure 1B, this earlier report found this approach to be ideal for identifying Sn^4+^ impurities on the samples. This evidence-based understanding enabled the usage of the Auger parameter analysis to determine Sn^4+^ impurities in our paper^1^ and has recently been validated by Li et al. for probing Sn-based perovskite nanocrystal surfaces.^15^ More information on the theoretical framework and best practices for the XPS analysis of Sn-based perovskites can be found in the related report by Wieczorek et al.^14^
  The AP relies on the energetic distance between spectroscopic features, rather than absolute binding energies, a concept known as internal charge referencing. This diminishes the issues related to C 1s charge referencing or charging. Internal charge referencing approaches have been known for decades but have been getting renewed attention in recent years.^16,17^ We have found internal charge referencing methods to be particularly useful for the analysis of semiconductor surfaces,^18–20^ including other types of metal halide perovskites.^21^ Likewise, both Biesinger and Greczynski et al. suggest the usage of Auger parameter analysis, the method we used in our original paper, as a solution to circumvent issues related to C 1s charge referencing.^10,22^

7. A. Dhingra suggests the usage of angle-resolved XPS measurements on the investigated core–shell nanocrystals “to get true physical insights into the surface stoichiometry”.
**Response:** The suggested usage of angle-resolved XPS by A. Dhingra is made redundant by the application of hard X-ray photoelectron spectroscopy (HAXPES) as presented in Figure 1f of our original paper. With HAXPES, a 3-fold increase in the probing depth is obtained due to the increased X-ray photon energy compared to conventional XPS.^23,24^ This allows us to see qualitative differences in the bulk and surface chemistry of our material. However, the complex core–shell structure of the nanocrystals prevents a robust quantitative analysis of the surface composition.

**Figure 1:**
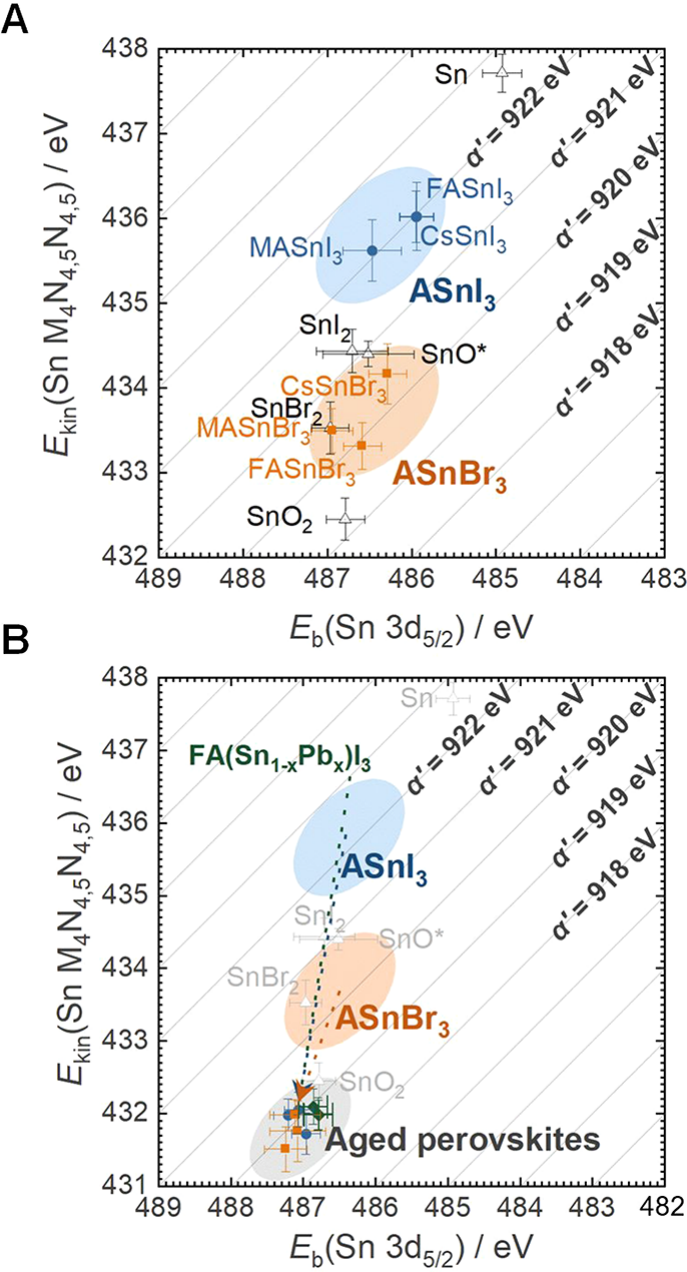
(A) Wagner plot depicting the Auger parameters α′ of ASnX_3_ perovskites for all permutations with A = methylammonium (MA), formamidinium (FA), and Cs, as well as X = I, Br. Reproduced or adapted from ref 14. Available under a CC-BY 4.0 license. Copyright 2023 A. Wieczorek, H. Lai, J. Pious, F. Fu, S. Siol. (B) Wagner plot depicting the shift of α′ for samples exposed to ambient conditions with resulting SnO_2_ formation on the surface. Reproduced or adapted from ref 14. Available under a CC-BY 4.0 license. Copyright 2023 A. Wieczorek, H. Lai, J. Pious, F. Fu, S. Siol.

In summary, we trust that this clarification addresses the concerns raised by A. Dhingra’s comment and demonstrates the rigor of our original paper. We hope it illuminates the reasoning behind our XPS analysis to a wide audience and fosters more productive, evidence-based discussions in the nanoscience and surface-science communities.
